# Effects of a Nurse-Led Web-Based Fall Prevention Program on Fall Risk and Fear of Falling Among Older Adults: A Randomized Controlled Trial

**DOI:** 10.3390/healthcare14172736

**Published:** 2026-08-27

**Authors:** Nur Sema Kaynar Demirel, Selda Secginli

**Affiliations:** 1 Department of Nursing, Faculty of Health Sciences, Van Yüzüncü Yıl University, Van 65080, Türkiye; nursemakaynar@yyu.edu.tr; 2 Department of Nursing, Faculty of Health Sciences, Atlas University, Istanbul 34403, Türkiye

**Keywords:** falls, fall risk, fear of falling, community-dwelling older adults, web-based fall prevention, nurse-led intervention

## Abstract

**Objectives**: This study aimed to evaluate the effects of a nurse-led Web-Based Fall Prevention Program (Web-FPP) on fall risk, fear of falling, fall-preventive behaviors, and the number of falls among community-dwelling older adults aged 65 years and older. **Methods**: A single-blind, stratified randomized controlled trial was conducted with 72 older adults (36 intervention, 36 control) in Türkiye. The Web-FPP was delivered over six weeks and included educational content, video-based exercises, and home safety strategies accessed through a web platform. Outcomes were assessed at baseline, immediately after the intervention, and at 3-month follow-up using the Timed Up and Go test (TUG), the Falls Efficacy Scale (FES), and the Falls Behavioural (FaB) Scale. **Results**: Compared with the control group, the intervention group showed significantly greater improvements in TUG performance, fear of falling, and fall-preventive behaviors, and these gains were maintained at three months (*p* < 0.05). No significant between-group difference was observed in the number of falls during the 3-month follow-up (*p* > 0.05). **Conclusions**: The findings suggest that nurse-led web-based fall prevention programs may represent an effective community-based strategy for reducing fall risk and fear of falling among older adults. Further studies with longer follow-up are needed to determine their effects on actual fall incidence.

## 1. Introduction

The global population is aging rapidly, with the proportion of older adults increasing significantly due to advancements in healthcare, declining birth rates, and longer life expectancy [1,2]. According to United Nations projections, global population ageing is expected to accelerate substantially, with the number of people aged 65 years and older projected to reach 2.2 billion by the late 2070s, surpassing the number of children younger than 18 years [3]. Türkiye is part of this global trend, with older adults making up 10.6% of the population in 2024 and projected to reach 17.9% by 2040 [4]. This demographic transformation brings significant health, social, and economic challenges, with falls emerging as one of the most common and serious problems among older adults [5,6]. One in three older individuals experiences a fall each year, and recurrent falls are frequent [7]. Approximately one in ten falls results in serious injuries such as fractures or head trauma, often leading to prolonged hospitalization, loss of independence, and psychological consequences like fear of falling [6,8,9]. Given their frequency and potentially severe outcomes, falls represent a major public health concern that profoundly impacts older adults’ quality of life by causing physical limitations, emotional distress, social isolation, and increased dependency.

Falls among older adults are rarely the result of a single cause. Instead, they typically occur due to a combination of biological, behavioral, and environmental risk factors—many of which are preventable [7]. As people age, these risk factors accumulate, increasing vulnerability to falls. More than half of falls occur at home and are often associated with environmental hazards [6]. In addition, age-related muscle loss is a major physiological contributor to fall risk. Given that falls are one of the leading causes of both fatal and non-fatal injuries in older adults, and significantly contribute to increasing healthcare costs, the need for effective and sustainable fall prevention strategies is evident [5]. In this context, the World Health Organization emphasizes the importance of prioritizing community-based interventions, as falls often lead to nursing home admissions [5].

In the 21st century, web-based interventions have emerged as promising tools for supporting the health and well-being of older adults, particularly due to their accessibility, cost-effectiveness, and adaptability [10,11]. Recent systematic reviews and meta-analyses indicate that ICT-based fall prevention interventions are effective in reducing fall risk and improving balance and falls efficacy among community-dwelling older adults [10]. These findings are further supported by individual intervention studies [12,13,14]. For instance, Moran et al. (2025) demonstrated a reduction in fall risk following a 12-week digital exercise program (“Strong Foundations”) targeting adults aged 60 and older [14]. Similarly, web-based fall prevention programs for specific clinical populations—such as individuals with multiple sclerosis—have shown potential benefits in reducing fall incidence [13]. Given the increasing use of digital health interventions in older populations, it is important that such programs are accessible and user-friendly. The Web-FPP was designed to support ease of use and usability, which are important considerations in technology acceptance [15]. Short videos, simple navigation, mobile accessibility, and weekly researcher contact were incorporated to reduce the cognitive and technical burden for older adults.

In the literature, fall prevention programs are generally categorized as either single-component (e.g., only exercise) or multicomponent (e.g., integrating exercise, education, and environmental modifications), with systematic reviews supporting the superior effectiveness of multicomponent approaches in reducing fall risk and improving fall-related outcomes [16,17,18,19]. However, while many countries have implemented such multicomponent interventions through digital platforms in community settings, studies in Türkiye have primarily taken place in institutional settings [20,21,22,23]. To the best of our knowledge, web-based fall prevention programs specifically targeting community-dwelling older adults have not yet been evaluated in Türkiye. This gap highlights the need to develop and evaluate innovative, nurse-led digital interventions that can be integrated into community-based preventive care. Nurses play a central role in community-based health promotion and chronic disease prevention. Their continuous interaction with older adults places them in a unique position to deliver individualized education, promote behavior change, and support adherence to fall prevention strategies. Therefore, nurse-led digital interventions may provide an effective and sustainable approach for preventing falls among community-dwelling older adults. Accordingly, this randomized controlled trial aimed to evaluate the effects of a nurse-led Web-Based Fall Prevention Program (Web-FPP) on fall risk, fear of falling, fall-preventive behaviors, and the number of falls among community-dwelling older adults in Türkiye.

## 2. Materials and Methods

### 2.1. Study Design and Sample

**Figure 1:**
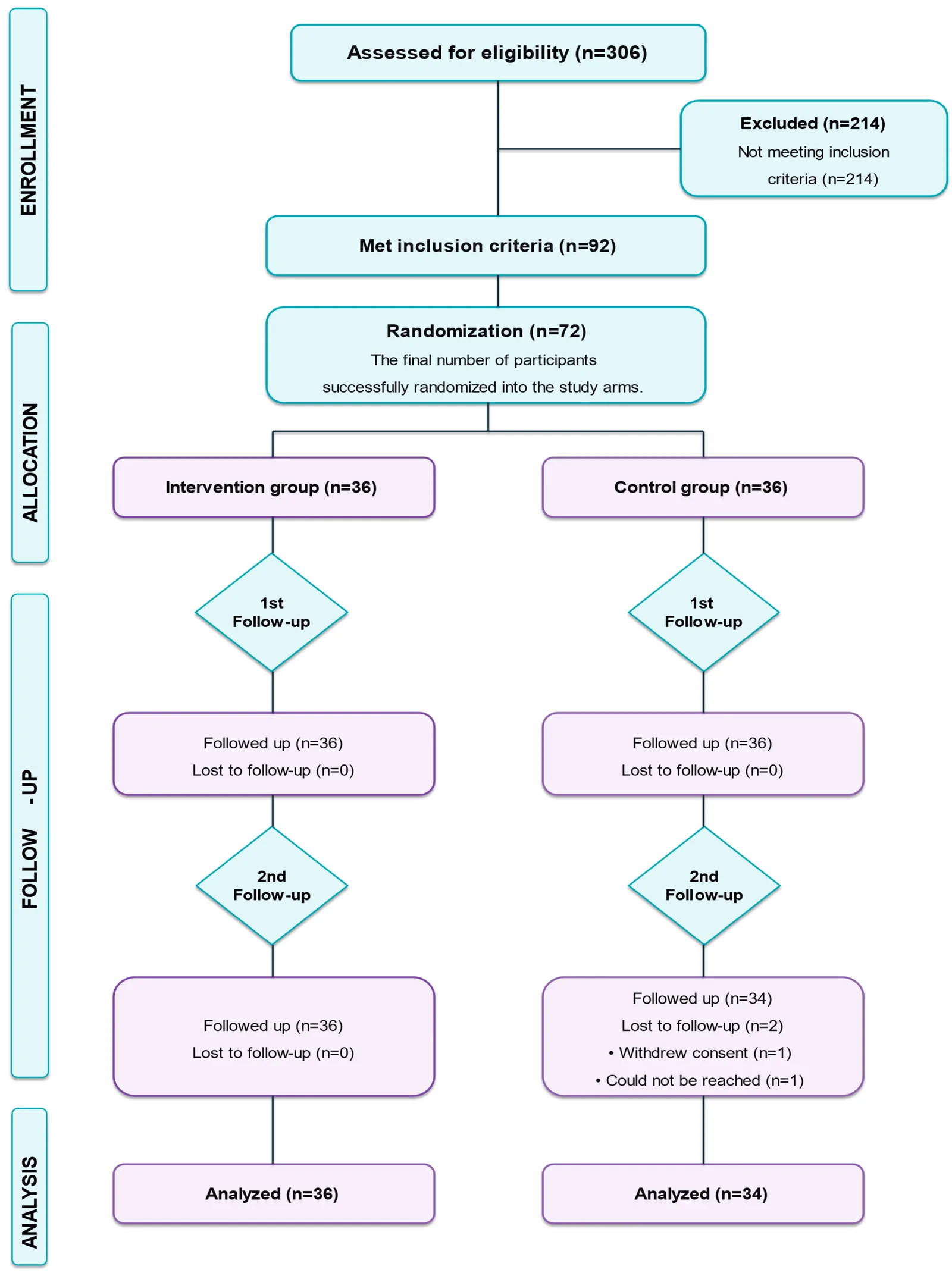
CONSORT flow diagram of the study.

This study was designed as a single-blind, pretest–posttest randomized controlled trial and was reported in accordance with the Consolidated Standards of Reporting Trials (CONSORT) guidelines (Figure 1). The study was registered on ClinicalTrials.gov on 2 June 2022 (NCT05406323) before participant enrollment, randomization, and initiation of the intervention. The primary outcomes were fall risk, assessed using the Timed Up and Go (TUG) test, and fear of falling, assessed using the Falls Efficacy Scale (FES). Secondary outcomes included fall-preventive behaviors, measured using the Falls Behavioural Scale (FaB), and the number of falls.

The target population of the study consisted of community-dwelling individuals aged 65 years and older who were registered in a neighborhood in a district of a province in western Türkiye. Participants were recruited through a neighborhood administrative office, which is frequently visited by older adults for administrative and social services. During the recruitment period, individuals attending the office were approached by the researcher and screened for eligibility based on the study criteria.

Between February and April 2022, a total of 306 individuals were screened for eligibility. Of these, 214 did not meet the inclusion criteria, leaving 92 eligible individuals. Following trial registration on ClinicalTrials.gov (2 June 2022; NCT05406323), 72 eligible individuals were enrolled according to the required sample size and randomly assigned to the intervention or control group. The sample size was determined using the “G*Power 3.1” program. Based on an effect size of 0.82 [22], with 80% power and an alpha of 0.01, the required sample size was calculated as 72 participants. Using stratified randomization, 36 participants were allocated to each group.

Participants were eligible for inclusion if they met the following criteria: (a) being a community-dwelling adult aged ≥65 years; (b) having internet access; (c) being able to use a computer, tablet, or smartphone and to communicate verbally and visually; (d) a Timed Up and Go Test score of ≥12 s; and (e) no contraindications to exercise (e.g., stroke, Parkinson’s disease, uncontrolled arrhythmia, severe cardiovascular or musculoskeletal disorders, or a recent hip/knee prosthesis within the past 6 months). Exclusion criteria included illiteracy, being bedridden, visual impairment, and moderate to severe cognitive impairment.

### 2.2. Randomization and Blinding

Participants were randomly assigned to either the Intervention Group (IG) or the Control Group (CG) using stratified randomization to ensure a balanced distribution of key risk factors between groups. The strata were defined based on age and fall history: (1) individuals aged 65–74 with a history of falls in the past year; (2) individuals aged 65–74 without a history of falls; (3) individuals aged 75 years and older with a history of falls; and (4) individuals aged 75 years and older without a history of falls. Following eligibility assessment and stratification, random allocation was performed within each stratum by an independent individual who was not involved in participant recruitment, intervention delivery, or outcome assessment using a computer-generated randomization sequence. The researcher was informed of group assignments only after the randomization procedure had been completed. Therefore, the researcher had no prior knowledge of participants’ subsequent group allocation during eligibility assessment.

Due to the nature of the intervention, blinding of both participants and the researcher was not feasible. The researcher conducted all outcome assessments and was therefore aware of group allocation. Statistical analyses were performed by an independent statistician who was blinded to group allocation. Accordingly, the study was conducted as a single-blind randomized controlled trial, with blinding limited to the statistician.

### 2.3. Web-Based Fall Prevention Program (Web-FPP)

#### 2.3.1. Development of the Web-FPP Website

The Web-FPP was delivered through a website specifically developed for this study. The website was designed by the researcher in collaboration with a computer engineer to provide a user-friendly and accessible interface. The website was developed using the Wix platform and was optimized for both desktop and mobile devices. The platform includes two interfaces: “User Profile” and “Control Panel.”

The User Profile interface was designed for participants in the IG. Upon logging in, users accessed information about the program, program content, and the contact page via the homepage. The program content was structured under three main headings: Education, Exercise, and Safe Home Environment. The Control Panel interface was developed for the researcher to manage the program. Through this panel, the researcher monitored participants’ video-viewing activity, completion of scheduled exercise sessions and the HSOF, and responses to the embedded questions. In addition, the researcher sent messages to individuals when necessary. Participant progress was monitored in real time via both the website and the Wix Owner application.

To improve usability for older adults, the website was designed with a simple, clear, and easy-to-navigate structure. Each participant was provided with a unique username and password to access the platform. Participants were also able to contact the researcher directly through the website whenever technical or program-related assistance was needed.

#### 2.3.2. Development of the Web-FPP

The nurse-led Web-FPP was developed as a multicomponent intervention to reduce falls, fall risk, and fear of falling among community-dwelling older adults. Accordingly, the Web-FPP included education, exercise, and home safety designed and delivered over six weeks. Prior to implementation, the researcher completed the Centers for Disease Control and Prevention (CDC)’s Stopping Elderly Accidents, Deaths & Injuries (STEADI) training to enhance applied knowledge of fall risk assessment and prevention.

##### Content of the Web-FPP

Education Component: The educational content of the Web-FPP was developed based on current evidence, international guidelines, and relevant literature [6,7,24,25]. The education program included seven topics: “Falls in Older Adults”, “Risk Factors for Falls”, “Fall Prevention Strategies: Exercise”, “Fall Prevention Strategies: Nutrition”, “Fall Prevention Strategies: Medication Use”, “Fall Prevention Strategies: Vision”, and “Fear of Falling.” Educational materials were prepared by the researcher using the Canva platform and were converted into short narrated videos. The duration of the videos ranged from 1 to 4 min. To support accessibility, all videos were optimized for mobile devices and presented through a simple, age-friendly interface to facilitate ease of use by older adults.

To enhance engagement and interactivity, each topic included 3 to 5 true/false questions integrated into the platform. A true/false format was selected to minimize response burden, reduce cognitive complexity, and support ease of use among older adults with varying levels of digital literacy. After responding, participants could view the correct answers and were provided with explanations when incorrect responses were given. This interactive structure aimed to reinforce key messages and promote active participation.

Exercise Component: One of the most effective components of fall prevention programs is exercise. Many recent studies have reported that exercise can reduce fall rates, fall risk, and fear of falling by improving strength and balance [2,16,18]. In this study, the exercise content was developed in collaboration with a physiotherapist and based on evidence-based components effective in fall prevention.

The program was inspired by selected movements from the Otago Exercise Program [26], but did not include the full set. In the original Otago program, exercises are individually tailored, progressively adjusted, and delivered under the supervision of a physical therapist over an extended period, and not all exercises are prescribed to every participant. Therefore, in the present study, a subset of exercises was selected and adapted for a standardized, web-based format. Exercises were selected based on their relevance to balance and lower-limb strengthening, safety for unsupervised home practice, and suitability for independent application by older adults. More complex components of the original Otago program requiring individualized prescription or direct supervision were not included, as the intervention aimed to provide a simple, accessible, and low-risk exercise model that could be delivered digitally without direct supervision. Accordingly, the exercise component consisted of eight standardized balance and strengthening exercises suitable for independent home practice. The balance exercises included sit-to-stand, heel raises, toe raises, knee bends, and sideways walking; the strengthening exercises included sideways leg raises, forward leg raises, and backward leg raises. Exercise instructions were delivered through an animated demonstration video and were also available on the platform as separate illustrated step-by-step instructions.

Participants were asked to perform the exercises three times per week starting from the first week; each movement began with five repetitions and was increased by one repetition each week to support gradual progression. Safety measures for the exercises were demonstrated in the training video to ensure correct and safe performance. Completion of the three scheduled weekly exercise sessions was monitored through the platform, while participants’ exercise experiences, difficulties, and any safety concerns were reviewed during weekly telephone follow-ups.

Safe Home Environment Component: Given that 82% of accidents among older adults occur at home, creating a safe home environment is essential to reduce fall risk [16,17]. To address this, participants viewed a 3-min educational video by The Royal Society for the Prevention of Accidents (RoSPA), highlighting common fall hazards in various parts of the home and offering strategies for prevention.

To support participants in identifying and addressing risks in their own homes, a researcher-developed Home Safety Observation Form (HSOF)—based on current guidelines and literature—was provided [27,28,29]. The HSOF was designed using a dichotomous (yes/no) response format to ensure simplicity and usability for older adults. This structure enabled participants to easily identify potential risks and receive tailored recommendations within the platform. Participants responded to yes/no questions, which generated automated, tailored on-screen recommendations for addressing identified home safety risks. Their responses were also reviewed by the researcher, who offered further guidance via phone calls when needed. The form was used solely for educational purposes and not as a data collection tool.

The detailed content and weekly structure of the Web-FPP are presented in Supplementary Figure S1.

### 2.4. Expert Review of the Web-FPP Content

The nine educational videos developed for this study were evaluated by 12 experts (8 academic nurses, 3 clinical nurses, and 1 physiotherapist) using the “Video Evaluation/Criteria” Form. The Video Evaluation/Criteria Form developed by the University of Vermont was translated into Turkish by the research team for use in this study. This form allowed for evaluation in terms of design (3 items) and content (5 items). Experts were asked to rate each item on a 5-point Likert scale, with a score of 3 or higher considered acceptable.

All items in both design and content categories were rated 4 or 5 by the experts. Based on the experts’ recommendations, minor revisions were made to improve the clarity and appropriateness of the educational materials. Specifically, the term “death” was replaced with “life-threatening”, the statement “take your medications properly” was revised to “take your medications at the right dose and at the right time”, and the number of slides in the “Fall Prevention Strategies: Nutrition” video was reduced.

### 2.5. Data Collection Tools

#### 2.5.1. Personal Information Form

Developed by the researchers based on the literature [21,22], this form included questions on participants’ socio-demographic and health-related characteristics (such as chronic disease, medication use, exercise).

#### 2.5.2. Timed Up and Go Test (TUG)

The TUG, developed by Podsiadlo and Richardson (1991), is frequently used to assess fall risk in older adults [30]. Participants were instructed to stand up from a standard chair, walk 3 m, turn around, walk back to the chair, and sit down. The time required to complete the test was recorded using a stopwatch. A time of ≥12 s indicates increased risk [31].

#### 2.5.3. Falls Efficacy Scale (FES)

The FES was developed by Tinetti et al. (1990) to evaluate an individual’s fear of falling while performing daily activities [32]. The validated Turkish version of the FES was used in this study [33]. This version includes 10 daily activities and is scored dichotomously as 1 = fear and 0 = no fear, with higher scores indicating greater fear of falling. Cronbach’s alpha was 0.89 in the Turkish version and 0.81 in this study.

#### 2.5.4. Falls Behavioural (FaB) Scale for the Elderly

The FaB Scale was developed by Clemson et al. (2003) to assess older adults’ fall-preventive behaviors and awareness [34]. It includes 30 items across 10 subscales, rated on a 4-point Likert scale (1 = Never, 2 = Sometimes, 3 = Often, 4 = Always). Higher scores reflect safer behaviors. Cronbach’s alpha was 0.90 in Turkish validation [35] and 0.75 in this study.

#### 2.5.5. Assessment of Falls

The number of falls was assessed through self-reports at baseline (falls in the past year), immediately post-intervention, and at the 3-month follow-up.

### 2.6. Data Collection

Data were collected after obtaining ethics committee approval and institutional permission through face-to-face interviews. Data were gathered at three time points: baseline, immediately post-intervention, and at three-month follow-up.

The Web-FPP was delivered via a web-based platform. A pilot study was conducted with four eligible individuals to assess the website’s usability and the clarity of the data collection tools. These individuals met the same eligibility criteria as participants enrolled in the main study; however, their sociodemographic characteristics were not formally compared with those of the main study sample. The pilot participants were not included in the main study.

#### 2.6.1. Intervention Group

Pre-intervention (Baseline):

Eligible participants aged 65+ were informed about the study, and baseline data were collected using the TUG, FES, FaB, and the number of falls.

Intervention:

Before initiating the Web-FPP, the intervention group attended face-to-face sessions on how to use the website via their own mobile devices. They were informed they could contact the researcher at any time via the website, phone, or SMS.

The intervention lasted six weeks and was designed to provide sufficient time for participants to complete all program components while maintaining feasibility and adherence. In addition, the six-week duration was considered appropriate to minimize participant burden and reduce attrition in community-dwelling older adults. Within this framework, participants followed the six-week program. They were asked to watch nine short videos (1–4 min) within the first two weeks. All content remained accessible throughout the program. The researcher monitored video engagement, completion of scheduled exercise sessions (minimum 3 days/week), and HSOF completion through the website’s control panel. Intervention fidelity was supported through this monitoring system, together with weekly reminder messages and telephone follow-ups. Weekly telephone follow-ups were conducted to monitor adherence, provide individualized feedback, address participants’ questions and exercise-related challenges, review home safety responses, and inquire about any falls or injuries related to the exercises. No exercise-related falls or injuries were reported. Access to the website was discontinued after the six-week intervention.

Post-intervention:

Immediately after the intervention and at the three-month follow-up, data were recollected via face-to-face interviews using the same tools: TUG, FES, FaB, and the number of falls.

#### 2.6.2. Control Group

The control group completed data collection at the same three time points using identical tools. No intervention was administered. After data collection, they were given access to the Web-FPP content for one month.

Throughout the study, participants in both groups continued to have access to routine health services as needed. No additional fall-prevention intervention was provided outside the study protocol.

### 2.7. Statistical Analysis

Data were analyzed using IBM SPSS Statistics version 20.0 (IBM, Armonk, NY, USA). Descriptive statistics included the mean, standard deviation, minimum–maximum, frequency, and percentage. Normality was assessed via the Kolmogorov–Smirnov and Shapiro–Wilk tests, supported by skewness–kurtosis values and histogram plots. Scale reliability was evaluated using Cronbach’s alpha. Independent samples *t*-test, Pearson’s Chi-square test, and Fisher–Freeman–Halton Exact tests were used to compare baseline and post-intervention data between groups. Repeated-measures ANOVA was used to evaluate changes in continuous outcomes across the three assessment time points and to examine whether these changes differed between the intervention and control groups. Estimated marginal means were used for between-group comparisons at each assessment point, with mean differences reported alongside 95% confidence intervals and Bonferroni-adjusted *p*-values; Bonferroni adjustment was also applied to within-group pairwise comparisons across time. The assumption of sphericity was evaluated using Mauchly’s test, and Greenhouse–Geisser corrections were applied when the assumption was violated. Statistical significance was set at *p* < 0.05.

Missing data analysis showed that two participants in the control group were lost to follow-up at the 3-month assessment (2.8%). According to Tabachnick and Fidell (2001), missing data rates below 5% are unlikely to substantially bias statistical results [36]. Therefore, no imputation procedure was applied, and the primary analyses were performed using complete-case analysis. In addition, a sensitivity analysis was performed using linear mixed-effects models to assess the robustness of the findings in the presence of missing follow-up data. The models included group, time, and group × time as fixed effects and incorporated all randomized participants with available observations.

### 2.8. Ethical Considerations

Ethical approval was obtained from an institutional ethics committee prior to participant recruitment (Approval Date: 9 December 2020; Decision No: 2020-199). Written and verbal informed consent was obtained from all participants before enrollment in the study. This study was conducted in accordance with the ethical principles of the Declaration of Helsinki.

## 3. Results

### 3.1. Descriptive Characteristics

A total of 72 older adults were randomized into the intervention and control groups. Two participants in the control group were lost to follow-up at the 3-month assessment. At baseline, there were no significant differences between the intervention and control groups in terms of sociodemographic or health characteristics (all *p* > 0.05) (Table 1).

Regarding intervention engagement, review of website usage records showed that 26 of the 36 participants (72.2%) viewed all nine intervention videos. Individual video-viewing rates ranged from 80.6% to 100%, with both the exercise and home-safety videos viewed by all participants.

### 3.2. Primary Outcomes

#### 3.2.1. Timed Up and Go (TUG) Test

A significant between-group difference was observed for TUG performance across the three assessment points (F = 34.330, *p* < 0.001, η^2^ = 0.506), indicating a large effect. The intervention group demonstrated marked improvements from baseline to post-intervention that were maintained at the 3-month follow-up, whereas the control group showed no significant change over time. Bonferroni-adjusted within-group pairwise comparisons showed significant improvements from baseline to post-intervention (MD = 1.74, *p* < 0.05) and from baseline to the 3-month follow-up (MD = 0.842, *p* < 0.05). A significant difference was also observed between the post-intervention and follow-up assessments (MD = −0.905, *p* < 0.05), while no significant within-group differences were observed in the control group (Table 2). Between-group comparisons based on estimated marginal means showed no significant difference in TUG performance at baseline (*p* > 0.05). At post-intervention, the estimated mean difference between the intervention and control groups was −3.618 (95% CI: −4.620 to −2.615; *p* < 0.001), and the corresponding difference at the 3-month follow-up was −1.984 (95% CI: −3.073 to −0.894; *p* < 0.001).

#### 3.2.2. Fear of Falling (FES)

A significant between-group difference was observed for FES scores across the three assessment points (F = 27.740, *p* < 0.001, η^2^ = 0.453), indicating a large effect. Fear of falling decreased significantly in the intervention group following the Web-FPP and remained lower at the 3-month follow-up. Bonferroni-adjusted pairwise comparisons showed significant reductions from baseline to post-intervention (MD = 0.712, *p* < 0.05) and from baseline to the 3-month follow-up (MD = 0.554, *p* < 0.05), as well as a significant difference between the post-intervention and follow-up assessments (MD = −0.158, *p* < 0.05). In contrast, fear of falling increased significantly over time in the control group (Table 2). Between-group comparisons based on estimated marginal means showed no significant difference in FES scores at baseline (*p* > 0.05). At post-intervention, the estimated mean difference between the intervention and control groups was −2.444 (95% CI: −3.393 to −1.496; *p* < 0.001), and the corresponding difference at the 3-month follow-up was −2.539 (95% CI: −3.527 to −1.551; *p* < 0.001).

### 3.3. Secondary Outcomes

#### 3.3.1. Falls Behavioural (FaB) Scale

A significant between-group difference was observed for FaB scores across the three assessment points (*F* = 177.559, *p* < 0.001, η^2^ = 0.857), indicating a large effect. Mean FaB scores increased from baseline to post-intervention and were maintained at the 3-month follow-up. Bonferroni-adjusted within-group pairwise comparisons showed significant increases in FaB scores from baseline to post-intervention (MD = −0.191, *p* < 0.05) and from baseline to the 3-month follow-up (MD = −0.190, *p* < 0.05), whereas no significant difference was observed between the post-intervention and follow-up assessments (MD = 0.002, *p* > 0.05). No significant within-group changes were observed in the control group (Table 3). Between-group comparisons based on estimated marginal means showed no significant difference in FaB scores at baseline (*p* > 0.05). The estimated mean difference between the intervention and control groups was 0.265 (95% CI: 0.159 to 0.371; *p* < 0.001) at post-intervention and 0.237 (95% CI: 0.134 to 0.340; *p* < 0.001) at the 3-month follow-up.

#### 3.3.2. Number of Falls

Mean fall counts decreased slightly over time in both groups. No significant between-group difference was observed in the number of falls across the three assessment points (*F* = 0.404, *p* = 0.669, η^2^ = 0.004), indicating a very small effect. Within-group changes over time did not reach statistical significance in either the intervention group (*F* = 3.065, *p* = 0.053) or the control group (*F* = 0.950, *p* = 0.392) (Table 3). Between-group comparisons based on estimated marginal means showed no significant difference in the number of falls at baseline (*p* > 0.05). No significant between-group differences were observed at post-intervention (MD = −0.029, 95% CI: −0.086 to 0.028; *p* = 0.307) or at the 3-month follow-up (MD = −0.033, 95% CI: −0.157 to 0.092; *p* = 0.602).

Sensitivity analyses using linear mixed-effects models yielded results consistent with the primary analyses. Significant group × time interactions were observed for TUG (*F* = 36.537, *p* < 0.001), FES (*F* = 28.005, *p* < 0.001), and FaB (*F* = 121.899, *p* < 0.001), indicating that the main findings remained robust when all available observations from randomized participants were included.

## 4. Discussion

This randomized controlled trial evaluated the effectiveness of the Web-FPP on fall-related outcomes among community-dwelling older adults. The intervention significantly improved fall risk, fear of falling, and fall-preventive behaviors, with these effects generally maintained at the 3-month follow-up. Although the number of falls did not differ significantly between groups, the findings support the potential role of brief, nurse-led web-based fall prevention programs in improving modifiable fall-related outcomes. Given the relatively short follow-up period, the absence of a significant reduction in fall incidence was not unexpected.

For fall risk, the intervention group showed significant reductions over time, consistent with previous research. For example, Moran et al. (2025) demonstrated a reduction in fall risk following a 12-week digital exercise program (“Strong Foundations”) targeting adults aged 60 and older [14]. Similarly, Lytras et al. (2022) observed a reduction in fall risk after a 6-month video-based Otago program delivered during the COVID-19 pandemic [37]. Although many existing studies employed longer intervention durations, the present results indicate that even relatively short, structured, and progressive exercise delivered via a web platform can meaningfully reduce fall risk. These findings are consistent with studies reporting that technology-supported exercise programs can enhance fall-related functional parameters among older adults [14,37], suggesting that brief digital interventions may be a feasible and scalable option, particularly in settings with limited access to in-person services. The reduction in fall risk may also be explained by the multicomponent structure of the Web-FPP, which combined balance and strengthening exercises with education and home safety strategies rather than relying on exercise alone. From a clinical perspective, the 3.5-s reduction in mean TUG time from baseline (14.5 s) to post-intervention (11.0 s) exceeded minimal detectable change (MDC) values of 2.08–3.00 s reported in studies of community-dwelling adults and older adults at high risk of falling, suggesting that the immediate improvement was unlikely to reflect measurement variability alone [38,39]. However, because reported MDC values vary across populations and measurement conditions, this finding should be interpreted cautiously.

In the present study, in addition to fall risk, fear of falling also significantly decreased in the intervention group compared to the control group following the intervention. Although a clinically important change threshold has not been established for the specific FES version used in this study, the observed reduction indicates that participants reported fear of falling across fewer daily activities. Therefore, these findings should be interpreted as improvements in self-reported fear of falling rather than against a predefined clinical threshold. This finding is supported by several studies indicating that multicomponent or exercise-based interventions can effectively reduce fear of falling. For example, Lytras et al. (2022) and Gholamzadeh et al. (2021) found that video-supported Otago programs and the “Stepping On” program reduced fear of falling in older adults [37,40]. However, some studies did not report significant changes in fear of falling following similar interventions. For instance, Bjerk et al. (2019) reported that a 12-week home-based fall prevention program did not significantly affect fear of falling at 3 months among Norwegian older adults receiving home care services [41]. These differences in outcomes may reflect variations in intervention content, delivery methods, and target populations. In the present study, the observed reduction in fear of falling may be attributed to the integration of educational components addressing fear, fall-related risk factors, and preventive strategies, along with regular physical activity and home safety measures. The weekly researcher contact may also have contributed to reassurance, motivation, and perceived support, which are important for older adults who experience activity restriction due to fear of falling. The practical relevance of addressing fear of falling is further supported by recent evidence showing that it is associated with older adults’ perceived safety and quality of life [42]. Given their close contact with older adults and their role in preventive care, nurses are well positioned to assess and manage fear of falling in this population [43].

The Web-FPP also led to significant increases in fall-preventive behaviors, with improvements observed across all FaB subscales except Displacing Activities, which showed no significant change. Although an established clinically important change threshold is not available for the FaB Scale, the observed improvement is practically relevant because the scale assesses everyday fall-preventive behaviors and awareness. Therefore, the increase in FaB scores can be interpreted as a favorable behavioral change rather than against a predefined clinical threshold. These findings are consistent with previous studies using the same scale, which also reported improvements in fall-related behaviors following intervention programs and nurse-led interventions [40,44,45]. For example, Tiedemann et al. (2021) reported that a 7-week community-based intervention using the Stepping On program in Australia led to significant improvements in fall-related behaviors at 6-month follow-up among adults aged 65 and older [45]. In this study, the lack of improvement in Displacing Activities, which reflects avoidance of outdoor activities under adverse conditions, may be explained by the limited emphasis on this component within the program. Overall, the results suggest that the nurse-led Web-FPP effectively promoted safer fall-related behaviors among older adults, reinforcing the role of nurses in community-based fall prevention efforts. These improvements may be related to the multicomponent nature of the intervention, which combined education, exercise practice, and home safety recommendations to support fall-preventive behaviors. 

Despite improvements in fall risk, fear of falling, and fall-preventive behaviors, no significant between-group difference was observed in the number of falls. This finding is not unexpected given the short duration of the intervention and follow-up in the present study. Evidence suggests that reductions in fall incidence are difficult to detect over limited observation periods because falls are episodic and multifactorial events; notably, even longer and well-designed interventions may fail to demonstrate early reductions in fall rates [46]. Moreover, current guidelines indicate that at least 12 months of monitoring is typically required to capture sufficient fall events and detect meaningful differences [47]. Given the short follow-up period, the lack of significant differences in fall incidence is not unexpected. Future studies with longer follow-up periods and larger samples are warranted to determine whether improvements in fall risk and fall-preventive behaviors translate into sustained reductions in fall incidence.

Another important aspect of this study is its demonstration of the feasibility and effectiveness of a nurse-led, web-based intervention. Nurses routinely assess fall risk, provide health education, and guide older adults in preventive strategies, positioning them well to deliver structured digital fall prevention content [48]. In primary and community care settings, particularly where access to physiotherapists or rehabilitation services is limited, nurse-led digital interventions may serve as a practical and resource-efficient approach to supporting fall prevention. The web-based format may also increase continuity of preventive education by allowing older adults to revisit content at home and receive follow-up support without requiring repeated face-to-face visits. However, the effectiveness of such programs depends not only on clinical content but also on usability, perceived usefulness, and perceived ease of use, which are central determinants of technology acceptance [15]. Therefore, initial orientation, simple navigation, mobile compatibility, and ongoing support appear to be important features for digital fall prevention programs targeting older adults. The web-based format may also facilitate broader implementation by enabling standardized intervention content to be delivered to older adults beyond traditional face-to-face settings. However, scalability and cost-effectiveness were not evaluated in the present study and should be examined in future implementation and economic evaluations.

Taken together, these findings contribute to the growing evidence supporting digitally delivered fall prevention programs for community-dwelling older adults. The observed improvements in fall-related outcomes indicate that brief web-based interventions can complement traditional face-to-face fall prevention programs. At the same time, digital interventions should be implemented with attention to equity. Older adults’ engagement with digital health tools can be influenced by digital health literacy, access to electronic devices, previous technology experience, and social support [49]. In this study, the requirement for internet access and basic device use may have selected a relatively functional and digitally capable group of older adults. Therefore, future implementations should consider caregiver-assisted models, blended face-to-face and digital delivery, and simplified user-centered designs to reduce digital exclusion. Because the Web-FPP was evaluated as an integrated multicomponent intervention, the independent contribution of each component to the observed effects cannot be determined. Future research should therefore consider designs that can distinguish the contributions of individual intervention components, along with longer follow-up periods, objective adherence monitoring, seasonal or environmental considerations, and user-centered approaches to enhance engagement, usability, and long-term effectiveness.

### Strengths and Limitations

This single-blinded, randomized controlled trial was methodologically rigorous and preregistered at ClinicalTrials.gov, enhancing its credibility. To our knowledge, this study represents the first nurse-led, web-based, multicomponent fall prevention program developed for community-dwelling older adults in Türkiye. Another strength is that the intervention combined evidence-informed educational content, video-based exercise, and home safety strategies within a single digital platform, supported by weekly follow-up and researcher feedback.

Several limitations should be considered when interpreting the findings of this study. First, the 3-month follow-up period may have been insufficient to detect significant differences in fall incidence, as falls are multifactorial and influenced by seasonal factors. Second, falls were assessed by self-report, which may have introduced recall bias. Third, because the researcher delivered the intervention and conducted all outcome assessments, blinding of the outcome assessor was not feasible. This may have introduced a risk of detection bias, particularly for assessor-dependent outcomes such as the TUG. Fourth, the intervention group received regular follow-up, reminders, and researcher feedback throughout the intervention period. Therefore, the additional researcher contact associated with the intervention may have contributed to the observed effects, representing a potential source of performance bias. Fifth, although the sample size met the a priori power calculation, recruitment from a single geographical area may limit the generalizability of the findings. Larger multicenter studies may provide more precise estimates of less frequent outcomes such as falls and enhance external validity. Sixth, although participants received instruction on website use, digital proficiency was not assessed using a standardized tool, which may have affected engagement with the intervention. In addition, requiring internet access and basic device use may have resulted in the inclusion of relatively functional and digitally capable older adults. The relatively young age of the sample (mean age approximately 68 years) should also be considered when interpreting the findings. Consequently, these characteristics may limit the generalizability of the findings to frailer older adults, adults of more advanced age, or those with limited digital literacy. Finally, although access to the exercise content and completion of the HSOF were monitored through website records and weekly follow-ups, the actual performance of the prescribed exercises and implementation of the recommended home-safety modifications could not be objectively verified. Future studies should evaluate adapted or caregiver-supported digital delivery models to improve accessibility and reduce digital inequities among older adults.

## 5. Conclusions

This study suggests that the nurse-led Web-FPP, which combined education, exercise, and home safety strategies, may be effective in reducing fall risk and fear of falling and in promoting fall-preventive behaviors among older adults. Although no significant change was observed in the number of falls, improvements in other key outcomes highlight the potential of web-based multicomponent interventions for fall prevention. These findings are particularly relevant given that many risk factors for falls are modifiable and preventable. The Web-FPP offers an accessible model that may be adapted for older adults from diverse backgrounds, particularly those with limited access to face-to-face care. Future studies should examine long-term effectiveness and cost-effectiveness. Integrating fall risk screening into primary care and involving public health nurses in prevention strategies may further reduce the burden of falls at the population level.

## Figures and Tables

**Table 1:** Sociodemographic and health characteristics of participants.

Variables	Intervention (*n* = 36) *n* (%)	Control (*n* = 36) *n* (%)	Test Statistic	*p*-Value
Age group			0.084 ^a^	0.772 *
65–69 years	29 (80.6)	28 (77.8)		
70–77 years	7 (19.4)	8 (22.2)		
Mean ± SD (Min–Max)	67.7 ± 2.8 (65–75)	67.9 ± 3.1 (65–77)	−0.198 ^b^	0.843 *
Gender			0.223 ^a^	0.637 *
Female	20 (55.6)	17 (47.2)		
Male	16 (44.4)	19 (52.8)		
Marital status			0.000	1.000 *
Married	29 (80.6)	29 (80.6)		
Single	7 (19.4)	7 (19.4)		
Education level			2.007 ^c^	0.599 *
Primary Education	6 (16.7)	11 (30.6)		
Secondary Education	17 (47.2)	14 (38.9)		
High School	9 (25.0)	8 (22.2)		
Higher Education	4 (11.1)	3 (8.3)		
Chronic disease			0.296 ^a^	0.586 *
Present	28 (77.8)	26 (72.2)		
Not present	8 (22.2)	10 (27.8)		
Regular medication use			0.296 ^a^	0.586 *
Yes	28 (77.8)	26 (72.2)		
No	8 (22.2)	10 (27.8)		
Number of medications used				
None	8 (22.2)	10 (27.8)	4.063 ^c^	0.560 *
One medication	4 (11.2)	1 (2.6)		
Two medications	7 (19.4)	8 (22.2)		
Three medications	6 (16.6)	9 (25.0)		
Four medications	8 (22.2)	4 (11.2)		
Five or more medications	3 (8.4)	4 (11.2)		
Regular Exercise				
No	36 (100.0)	36 (100.0)		

Note: SD = Standard Deviation; ^a^ = χ^2^ Chi-square test; ^b^ = Independent samples *t*-test; ^c^ = Fisher’s Exact Test; * *p* > 0.05 indicates no significant difference.

**Table 2:** Comparison of primary outcome scores in the intervention and control groups.

Primary Outcomes		Intervention (*n* = 36) M (SD)	Control (*n* = 36) M (SD)	Between-Group Comparison (*F*/*p*)	Comparison	Mean Difference/*p*	η^2^
TUG	Baseline	14.5 (2.3)	14.5 (2.2)	34.330/<0.001 **	Baseline vs. Post	1.74/<0.05 *	0.506
	Post-intervention	11.0 (2.2)	14.6 (2.0)		Baseline vs. Follow-up	0.842/<0.05 *	
	3-month follow-up	12.7 (2.4)	14.7 (2.1)		Post vs. Follow-up	−0.905/<0.05 *	
	F/*p*	71.913/<0.001 **	0.145/0.866				
FES	Baseline	3.3 (2.6)	3.6 (2.4)	27.740/<0.001 **	Baseline vs. Post	0.712/<0.05 *	0.453
	Post-intervention	1.6 (1.4)	4.0 (2.5)		Baseline vs. Follow-up	0.554/<0.05 *	
	3-month follow-up	1.7 (1.5)	4.2 (2.6)		Post vs. Follow-up	−0.158/<0.05 *	
	F/*p*	39.496/<0.001 **	4.800/0.011				

Note: TUG = Timed Up and Go; FES = Falls Efficacy Scale; M = Mean; SD = Standard Deviation; *F* = *F* value from repeated measures ANOVA (Wilks’ Lambda Test), * *p* < 0.05, ** *p* < 0.001.

**Table 3:** Comparison of secondary outcome scores in the intervention and control groups.

Secondary Outcomes		Intervention (*n* = 36) M (SD)	Control (*n* = 36) M (SD)	Between-Group Comparison (*F*/*p*)	Comparison	Mean Difference/*p*	η^2^
FaB	Baseline	2.26 (0.21)	2.32 (0.25)	177.559/<0.001 **	Baseline vs. Post	–0.191/<0.05 *	0.857
	Post-intervention	2.64 (0.21)	2.33 (0.25)		Baseline vs. Follow-up	–0.190/<0.05 *	
	3-month follow-up	2.62 (0.21)	2.35 (0.23)		Post vs. Follow-up	0.002/>0.05	
	F/*p*	386.843/0.001 **	2.032/0.139				
Number of falls	Baseline	1.53 ± 0.73	1.53 ± 0.74	0.404/0.669 *	Baseline vs. Post	0.075/>0.05	0.004
	Post-intervention	1.31 ± 0.47	1.38 ± 0.49		Baseline vs. Follow-up	0.074/>0.05	
	3-month follow-up	1.28 ± 0.45	1.41 ± 0.50		Post vs. Follow-up	−0.001/>0.05	
	F/*p*	3.065/0.053	0.950/0.392				

Note: FaB = Falls Behavioural Scale; M = Mean; SD = Standard Deviation; *F* = *F* value from repeated measures ANOVA (Wilks’ Lambda Test), * *p* < 0.05, ** *p* < 0.001.

## Data Availability

The data presented in this study are available on request from the corresponding author. The data are not publicly available due to privacy and ethical restrictions regarding participant anonymity.

## Supplementary Materials

The following supporting information can be downloaded at https://www.mdpi.com/article/10.3390/healthcare14172736/s1, Figure S1: Detailed content and weekly structure of the Web-Based Fall Prevention Program (Web-FPP).

## Abbreviations

The following abbreviations are used in this manuscript:

Web-FPP	Web-Based Fall Prevention Program
CONSORT	Consolidated Standards of Reporting Trials
CDC	Centers for Disease Control and Prevention
STEADI	Stopping Elderly Accidents, Deaths & Injuries
TUG	Timed Up and Go Test
FES	Falls Efficacy Scale
FaB	Falls Behavioural Scale
HSOF	Home Safety Observation Form
IG	Intervention Group
CG	Control Group
MDC	Minimal Detectable Change
