# Supplementary material for: Effects of a Nurse-Led Web-Based Fall Prevention Program on Fall Risk and Fear of Falling Among Older Adults: A Randomized Controlled Trial
Source: Healthcare (Basel). 2026 Aug 27;14(17):2736. doi: 10.3390/healthcare14172736 (PMC13564696; doi:10.3390/healthcare14172736)
Supplement: Supplementary file 1 [file healthcare-14-02736-s001.zip › healthcare-4454274-supplementary.pdf]

Web-Based Fall Prevention Program (Web-FPP)

COMPONENT	WEEK 1	WEEK 2	WEEK 3	WEEK 4	WEEK 5	WEEK 6
**EDUCATION**<br>Health education videos	• **Falls in older adults**<br>• **Risk factors for falls**<br>• **Fall prevention strategies (exercise)**	• **Nutrition**<br>• **Medication use**<br>• **Vision**<br>• **Fear of falling**	**--------------------- No new education videos ---------------------**<br>*(Participants can rewatch the videos as needed)*			
**EXERCISE**<br>Balance and strengthening exercises video	**Learn the exercises**<br>Perform ≥3 days/week<br>5 repetitions of each movement	**6** repetitions of each movement	**7** repetitions of each movement	**8** repetitions of each movement	**9** repetitions of each movement	**10** repetitions of each movement
	**Perform the exercises ≥3 days/week throughout the program**					
**SAFE HOME ENVIRONMENT**<br>Safe home environment		Home safety environment video<br>Weekly review of home fall risks using the Home Safety Observation Form (HSOF)	**--- Weekly review of home fall risks using the Home Safety Observation Form (HSOF) ---**			

Notes

- Education component is delivered in Weeks 1–2.
- Exercise component is performed at least 3 days per week; repetitions are progressively increased from 5 to 10.
- Home safety risks are reviewed weekly using the Home Safety Observation Form (HSOF) from week 2 through week 6.
- Weekly telephone follow-up is provided throughout the program to support adherence and address questions.

HSOF: Home Safety Observation Form

Web-FPP: Web-Based Fall Prevention Program

Supplementary Figure S1. Detailed content and weekly structure of the Web-Based Fall Prevention Program (Web-FPP).
